# Warming and shifts in litter quality drive multiple responses in freshwater detritivore communities

**DOI:** 10.1038/s41598-024-61624-z

**Published:** 2024-05-15

**Authors:** Sandra Benavides-Gordillo, Angélica L. González, Mônica F. Kersch-Becker, Marcelo S. Moretti, Dieison A. Moi, Marcos P. M. Aidar, Gustavo Q. Romero

**Affiliations:** 1 Laboratório de Interações Multitróficas e Biodiversidade, Departamento de Biologia Animal, Instituto de Biologia, Universidade Estadual de Campinas (UNICAMP), CP 6109, Campinas, São Paulo 13083-862 Brazil; 2 Biology Department and Center for Computational and Integrative Biology, Rutgers, The State University of New Jersey, Camden, NJ USA; 3 Department of Entomology and Center for Chemical Ecology, Pennsylvania State University, University Park, PA 16802 USA; 4 Laboratory of Aquatic Insect Ecology, Universidade Vila Velha, Vila Velha, Espírito Santo 29102920 Brazil; 5 Plant Physiology and Biochemistry of Botany, Institute of Botany, CP 3005, São Paulo, 01061-970 Brazil

**Keywords:** Biodiversity, Climate-change ecology, Community ecology, Ecosystem ecology, Freshwater ecology, Tropical ecology

## Abstract

Aquatic detritivores are highly sensitive to changes in temperature and leaf litter quality caused by increases in atmospheric CO_2_. While impacts on detritivores are evident at the organismal and population level, the mechanisms shaping ecological communities remain unclear. Here, we conducted field and laboratory experiments to examine the interactive effects of changes in leaf litter quality, due to increasing atmospheric CO_2_, and warming, on detritivore survival (at both organismal and community levels) and detritus consumption rates. Detritivore community consisted of the collector-gathering *Polypedilum* (Chironomidae), the scraper and facultative filtering-collector Atalophlebiinae (Leptophlebiidae), and Calamoceratidae (Trichoptera), a typical shredder. Our findings reveal intricate responses across taxonomic levels. At the organismal level, poor-quality leaf litter decreased survivorship of *Polypedilum* and Atalophlebiinae. We observed taxon-specific responses to warming, with varying effects on growth and consumption rates. Notably, species interactions (competition, facilitation) might have mediated detritivore responses to climate stressors, influencing community dynamics. While poor-quality leaf litter and warming independently affected detritivore larvae abundance of Atalophebiinae and Calamoceratidae, their combined effects altered detritus consumption and emergence of adults of Atalophlebiinae. Furthermore, warming influenced species abundances differently, likely exacerbating intraspecific competition in some taxa while accelerating development in others. Our study underscores the importance of considering complex ecological interactions in predicting the impact of climate change on freshwater ecosystem functioning. Understanding these emergent properties contributes to a better understanding of how detritivore communities may respond to future environmental conditions, providing valuable insights for ecosystem management and conservation efforts.

## Introduction

Since the pre-industrial era, atmospheric CO_2_ concentration has risen from 280 ppm to over 410 ppm (IPCC, 2014). By the end of this century, it may surge to as much as 936 ppm as projected in the worst-case scenario (RCP 8.5 scenario)^1^. This increase in atmospheric CO_2_ can affect the structure and functioning of both terrestrial and aquatic ecosystems^2,3^. Previous studies have demonstrated that elevated CO_2_ can alter the chemistry and physiology of terrestrial plants, resulting in an increased photosynthesis rate, high leaf carbon content, reduced leaf N and P content, and increased leaf C:nutrient ratio^4,5^. Such changes in leaf nutrient content can influence leaf litter quality^6^. In fact, studies have shown that plant chemical and physical defenses remain active even after senescence (e.g.,^7^). Consequently, changes in leaf litter nutrient content and C:nutrient ratio can reduce leaf litter palatability and hinder litter processing by detritivores and microbial decomposers^2,8^. These changes can also impact the performance, fitness and survival of aquatic detritivores^9,10^, thereby modifying the detrital processing chain in detritivore communities.

In addition to changes in plant nutrient content, the increase in atmospheric CO_2_ is contributing to a rise in global temperatures. It is estimated that by 2100, under the “without mitigation scenario”, the atmospheric temperature will increase by 4 °C^1^. Given that biological rate processes are temperature-dependent, the temperature increase is anticipated to accelerate organismal metabolism^11^, leading to higher rates of consumption^12^ and growth^13^. This will shorten development times and reduce size at maturity^14^, causing cascading consequences at the community and ecosystem scales^15,16^. Asymmetric responses among species and functional groups to climate change can disrupt trophic interactions and alter top-down or bottom-up processes^17,18^. Alternatively, climate change may alter the strength of trophic interactions by enhancing, for instance, consumer-resource interactions due to an increase in metabolic demands unlikely to be additive or linear^19,20^, resulting in unexpected or counterintuitive outcomes^21,22^. Finally, climate change is a complex combination of stressors, and the synergies between them could amplify the effect of a single climate stressor.

The combined effects of leaf litter quality and warming can play an important role in shaping species interactions, community structure and ecosystem functioning^23–25^. Aquatic detritivores exhibit a rapid response to changes in leaf litter quality and temperature (e.g.,^10,26–28^. However, the effects of these two drivers can be antagonistic, making it challenging to predict their ultimate outcomes^10^. While most studies assessing the interactive effects of poor leaf litter quality and warming on detritivore performance have focused on organismal- or population-level responses^10,28–30^, little is known about the inter and intraspecific interactions occurring within and among trophic levels (e.g., interactions among shredders, scrapers, collector-gatherers). Within a trophic level, synergistic mechanisms, such as complementarity or facilitation, may ameliorate or enhance species responses to changes in leaf litter quality. For instance, in a mutualistic or facilitative interaction, if the benefactor species is negatively affected by an environmental stressor, the species benefiting from it via facilitation or complementarity may be indirectly vulnerable to that stressor^31,32^. Conversely, it has been hypothesized that the lack of high-quality leaf litter increases resource competition^33^, potentially exacerbated by warming. Understanding the emergent properties that arise from inter and intraspecific interactions significantly contribute to more accurately predicting the effects of climate change on aquatic detritivore communities.

Empirical evidence on the interactive effects of warming and leaf litter quality, driven by increased atmospheric CO_2_, on the performance and survival of aquatic detritivores is still limited (see^10,29^). To fill this gap, we tested the effects of leaf litter quality, achieved by experimentally increasing atmospheric CO_2_ during the plant development, and warming, at both organismal and community levels, using freshwater insects from distinct feeding groups (i.e., shredders, scrapers, collector-gatherers). Specifically, we investigated the independent and interactive effects of leaf litter quality and warming at the organismal level (i.e., growth rate, survival and food consumption) and at community level (i.e., relative abundance of emerging adults, larvae survival and food consumption). The organismal-level refers to “each feeding guild separately” (i.e., monoculture), while the community-level refers to the three feeding guilds in polyculture. The elevated atmospheric CO_2_ concentration had a significant impact on leaf litter quality in our experiment, i.e., it increased leaf toughness, lignin concentrations and lignin:nitrogen ratios (see “Materials and methods” section and Supplementary Material 1). Thus, we predicted that (i) reduced leaf litter quality would lead to lower organismal growth rate, decreased leaf consumption rate, and reduced survival rate, as well as the relative abundance of detritivores; (ii) functional feeding groups closely associated with leaf litter (e.g., shredders) would be more strongly affected by changes in leaf litter quality. Furthermore, we predicted that (iii) warming would decrease survival rate but increase leaf consumption rate, as higher temperatures are known to accelerate organismal metabolic rates^11^. An increased organismal metabolic rate could potentially boost leaf consumption to support elevated metabolism. Considering the potential antagonistic effects between poor leaf litter quality and warming on survival, growth rate and consumption rates, we predicted that (iv) leaf litter quality could ameliorate the effects of warming, thus influencing detritivores at both organismal and community level.

## Results

### Organismal-level responses

**Figure 1:**
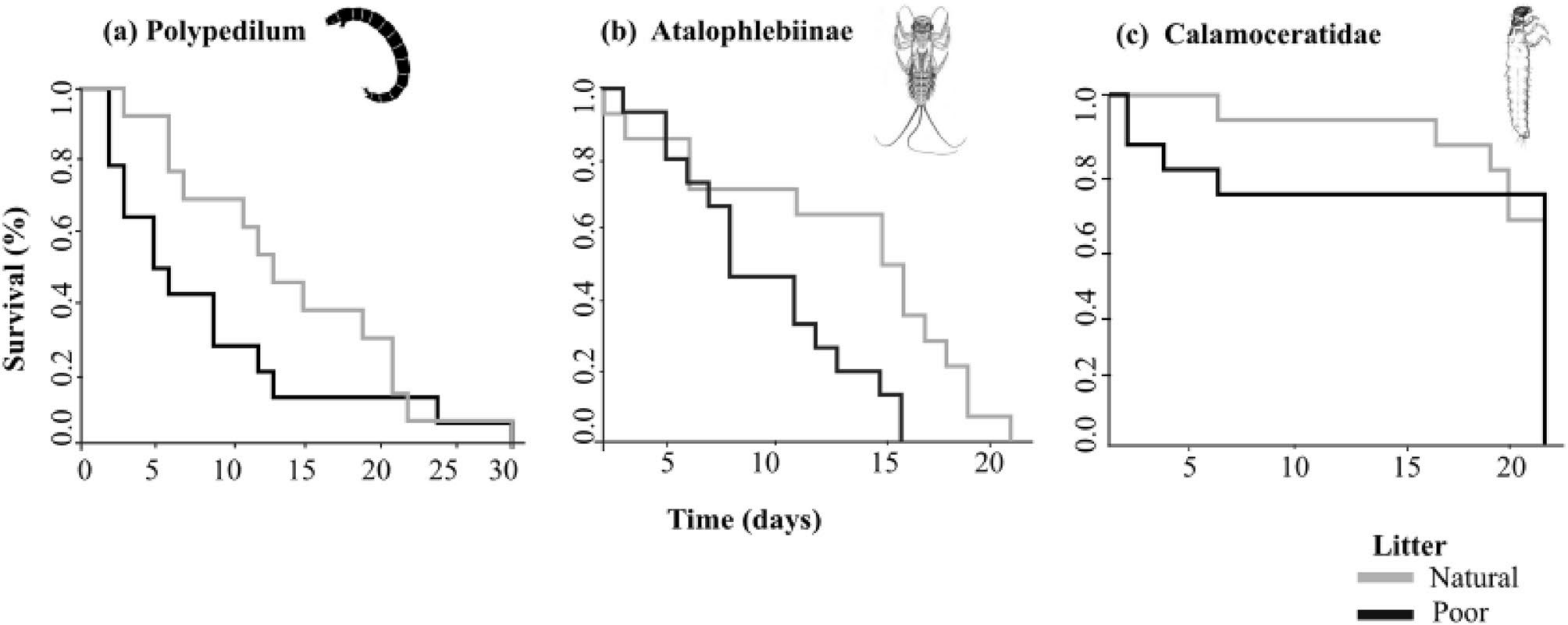
Effects of leaf litter quality (natural vs. poor) on survival rate of (**a**) *Polypedilum*, (**b**) Atalophlebiinae, and (**c**) Calamoceratidae.

Poor-quality leaf litter altered the survivorship of *Polypedilum* (χ2 = 4.85, *p* = 0.037) and Atalophlebiinae (χ2 = 5.39, *p* = 0.020), but not of Calamoceratidae (χ2 = 0.015, *p* = 0.995) (Fig. 1). Specifically, poor-quality leaf litter decreased the survival of *Polypedilum* by an average of 5 days compared to natural-quality leaf litter (Fig. 1A). However, after 30 days, the total number of dead *Polypedilum* larvae was similar between poor and natural-quality leaf litter (poor-quality litter = 14, natural quality litter = 13). The survival rate of Atalophlebiinae decreased by approximately 50% under poor-quality litter treatment by day 15 (Fig. 1B).

**Figure 2:**
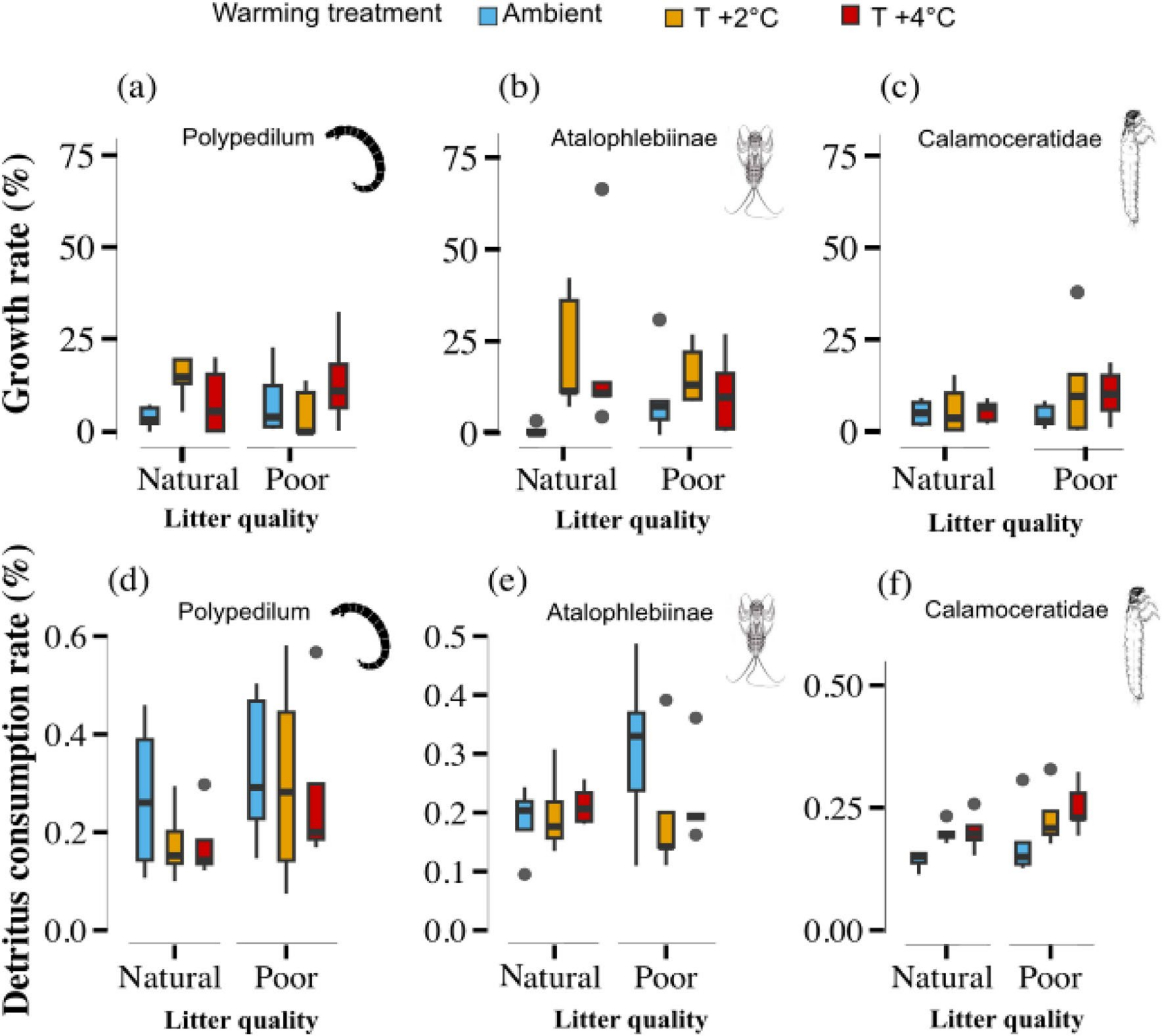
Effects of warming and leaf litter quality on detritivore growth rates (organismal level) and leaf consumption rates. Effects of warming (ambient, + 2 °C, and + 4 °C) and litter quality (natural and poor) on the growth rate (**a**–**c**) and leaf consumption rates (**d**–**f**) of three detritivore taxa: *Polypedilum,* Atalophlebiinae, and Calamoceratidae (n = 30). Box plots show the median (horizontal line), first and third quartile (rectangle), and outliers (isolated points).

An interactive effect between leaf litter quality and warming influenced *Polypedilum* growth rate but did not affect Calamoceratidae (Table 1, Fig. 2a,c). Elevating temperatures to + 2 °C enhanced *Polypedilum* growth by 60% (Fig. 2a). Nonetheless, poor-quality leaf litter attenuated the positive effects of warming, resulting in a decrease in *Polypedilum* growth rate (Fig. 2a). Conversely, changes in leaf litter quality and temperature independently influenced the growth rate of Atalophlebiinae (Table 1). Increasing temperature to + 2 °C boosted Atalophlebiinae growth by 20%, while poor-quality leaf litter increased it by 15% (Fig. 2b). Finally, increasing temperature to + 4 °C had no discernible impact on the growth rates of *Polypedilum* and Atalophlebiinae (Fig. 2a,b).

**Table 1:** Generalized least squares (GLS) results for organismal level experiment comparing the effects of temperature (Ambient vs. + 2 °C and ambient vs. + 4 °C), leaf litter quality (natural vs. poor), and their interaction (warming × litter quality) on the (i) growth rate of each detritivore taxa and (ii) their rate of leaf consumption.

Source of variation	Treatment								
	Litter poor			Temperature [T2]			Temperature [T4]		
	Estimates	CI	*P*	Estimates	CI	*P*	Estimates	CI	*P*
Growth rates									
* Polypedilum*	5.00	− 4.48 to 14.41	0.287	**10.60**	**4.47** to **16.72**	**0.002**	4.75	− 3.90 to 13.40	0.268
Atalophlebiinae	**10.66**	**0.26** to **3.12**	**0.023**	**19.40**	**4.53 ** to ** 34.27**	**0.013**	13.40	− 9.91 to 37.71	0.247
Calamoceratidae	− 0.87	− 5.02 to 3.29	0.671	1.05	− 5.87 to 7.97	0.756	0.57	− 3.46 to 4.60	0.773
Consumption rates									
*Polypedilum*	0.02	− 0.16 to 0.21	0.783	− 0.08	− 0.23 to 0.07	0.291	0.00	− 0.16 to 0.17	0.970
Atalophlebiinae	− 0.02	− 0.24 to 0.19	0.843	− 0.04	− 0.41 to 0.32	0.808	0.08	− 0.14 to 0.31	0.459
Calamoceratidae	0.02	− 0.08 to 0.12	0.668	**0.05**	− **0.02** to **0.09**	**0.003**	0.05	− 0.01 to 0.12	0.079

Source of variation	Treatment								
	Litter poor × Temperature [T2]			Litter poor × Temperature [T4]			*R*^2^		
	Estimates	CI	*P*	Estimates	CI	*P*			
Growth rates									
*Polypedilum*	− **14.30**	− **26.95 ** to − **1.66**	**0.028**	0.18	− 18.90 to 19.26	0.984	0.222		
Atalophlebiinae	− 13.73	− 31.94 to 4.48	0.133	− 15.38	− 40.98 to 10.42	0.232	0.196		
Calamoceratidae	7.84	− 8.19 to 23.86	0.323	5.32	− 2.82 to 13.36	0.190	0.169		
Consumption rates									
*Polypedilum*	− 0.00	− 0.21 to 0.21	0.985	− 0.04	− 0.27 to 0.19	0.713	− 0.018		
Atalophlebiinae	0.03	− 0.41 to 0.36	0.855	− 0.12	− 0.41 to 0.17	0.400	0.011		
Calamoceratidae	0.01	− 0.11 to 0.12	0.925	0.03	− 0.10 to 0.16	0.643	0.358		

The detritivore taxon were analyzed separately, with growth rate and leaf consumption rate corresponding to each taxon individually. Significant values are shown in bold.

Finally, leaf litter quality and warming did not affect leaf litter consumption by *Polypedilum* and Atalophlebiinae (Table 1, Fig. 2d,e). While leaf litter quality did not affect the consumption rate by Calamoceratidae, warming did (Table 1). Specifically, at + 2 °C, the consumption rate by Calamoceratidae increased by 10%, although no significant differences were observed at + 4 °C (Fig. 2f).

### Community-level experiment

The MANOVA test revealed significant effects of leaf litter quality on the abundance of live larvae of detritivore taxa (pillai = 0.445, *p* = 0.036). There were no significant effects of warming on the abundance of live larvae. In addition, leaf litter quality-by-warming interaction significantly affected the abundance of emerging adults (pillai = 0.375, *p* = 0.029).

**Figure 3:**
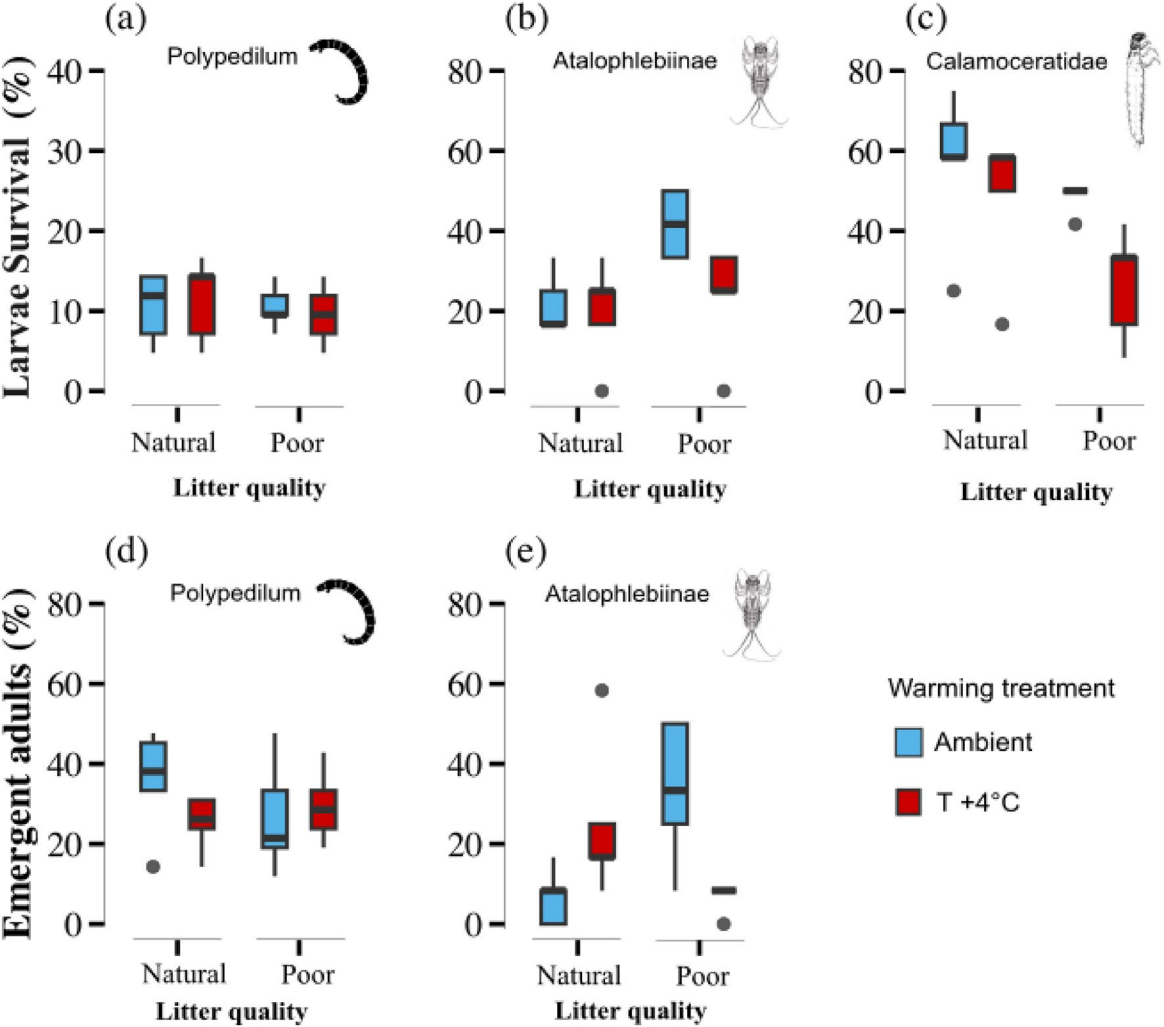
Effects of warming and leaf litter quality on individuals’ numbers and emergent adults of detritivore taxon in mixtures (community level). Effects of warming (ambient and + 4 °C) and litter quality (natural and poor) on the relative abundance [percentage of individuals alive (**a**–**c**) and emergent adults (**d**, **e**)] of three detritivore taxa: *Polypedilum,* Atalophlebiinae, and Calamoceratidae living in mixture experiment (n = 20). Box plots show the median (horizontal line), first and third quartile (rectangle), and outliers (isolated points).

Taxon-specific individual responses showed that leaf litter quality and warming did not affect the relative abundance of live larvae and the emergence of adults of *Polypedilum* (Table 2, Fig. 3a,d). However, contrary to predictions, leaf litter quality doubled the relative abundance of live nymphs of Atalophlebiinae (Fig. 3b). The leaf litter quality-by-warming interaction affected the relative abundance of emerging adults of Atalophlebiinae (Table 2, Fig. 3e). Poor quality litter increased the relative abundance of emerging adults by 20%, while warming increased it by 10% (Fig. 3e). However, at elevated temperatures, poor-quality leaf litter strongly decreased the relative abundance of emerging adults (Fig. 3e). Finally, poor quality leaf litter decreased the relative abundance of live larvae of Calamoceratidae by 15%, while warming independently decreased it by 25% (Fig. 3c). It is worth noting that no adults of Calamoceratidae were retrieved, suggesting that neither poor-quality leaf litter nor warming had significantly affected their emergence.

**Table 2:** Linear mixed effects models (LME) results for the community level experiment comparing the effect of temperature (Amb vs. + 4 °C), litter quality (natural vs. poor) and their interaction (warming × litter quality) on the (i) percentage of detritivores alive, (ii) percentage of adult emergence, (iii) rate of leaf consumption by the entire community.

Source of variation	Treatment					
	Litter quality		Temperature (T)		LQ × T	
	χ^2^	*p*	χ^2^	*p*	χ^2^	*p*
Detritivores alive						
*Polypedilum*	0.00	1.000	0.10	0.751	0.27	0.602
Atalophlebiinae	**10**.**5**	**0**.**001**	0.073	0.786	3.67	0.055
Calamoceratidae	**4**.**49**	**0**.**034**	**11**.**16**	**0**.**0008**	1.00	0.317
Adult emergent						
*Polypedilum*	0.98	0.320	2.75	0.096	2.00	0.156
Atalophlebiinae	**9**.**39**	**0**.**002**	**4**.**44**	**0**.**035**	**13**.**37**	**0**.**0002**
Calamoceratidae	–	–	–	–	–	–
Leaf consumption						
Community	**25**.**4**	< **0**.**00**	3.01	0.082	**5**.**43**	**0**.**019**

Degrees of freedom: CO_2_ = 1, temperature = 2. Significant values are shown in bold.

**Figure 4:**
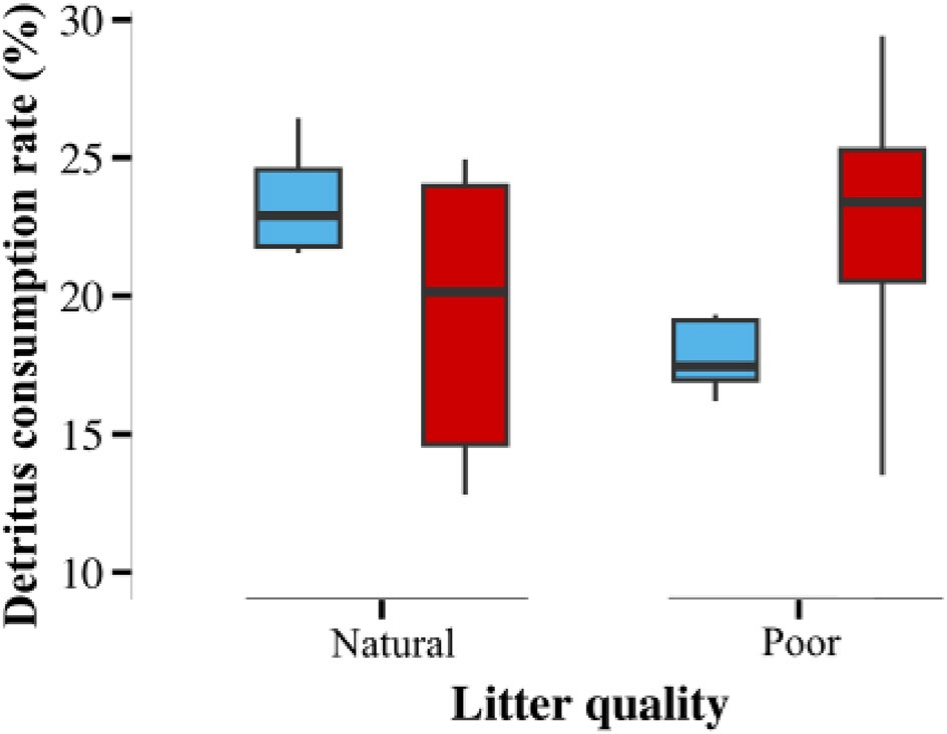
Effects of warming and leaf litter quality on leaf consumption by the detritivore community. Effects of warming (ambient and + 4 °C) and leaf litter quality (natural and poor) on overall leaf consumption by detritivore taxon living in the community experiment (n = 20). Box plots show the median (horizontal line), first and third quartile (rectangle), and outliers (isolated points).

As predicted, poor-quality leaf litter exhibited a 10% lower consumption compared to natural-quality leaf litter under ambient temperature. However, warming counteracted the effect of poor-quality leaf litter, leading to a 5% increase in the mass consumed under elevated temperatures (Table 2, Fig. 4). Contrary to predictions, warming decreased leaf consumption of natural-quality litter by 10% (Fig. 4).

**Figure 5:**
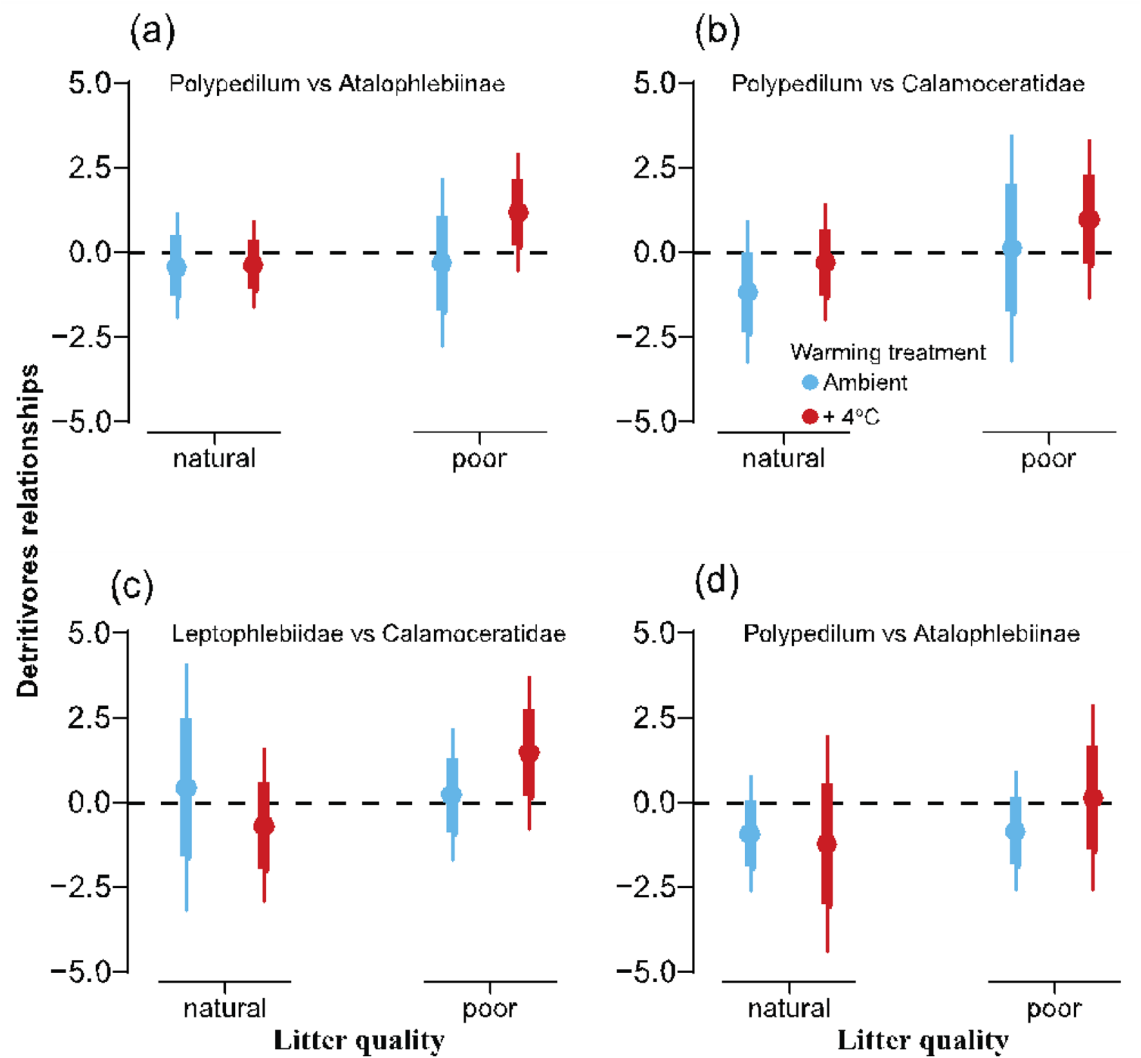
Effects of warming and leaf litter quality on the relationship between detritivore taxa in the community experiment. (**a**–**d**) Estimated coefficients for the relationship between detritivore taxa (*Polypedilum* vs. Atalophlebiinae, *Polypedilum* vs. Calamoceratidae*,* and Atalophlebiinae vs. Calamoceratidae) across warming x litter quality treatments. Points represent scaled estimates, thick lines represent 75% CIs, and thin lines represent 95% CIs.

Neither changes in litter quality nor warming had significant effects on the relationship between the percentage of individuals alive and emergent adults of detritivore taxa (*Polypedilum* vs. Atalophlebiinae, *Polypedilum* vs. Calamoceratidae*,* and Atalophlebiinae vs. Calamoceratidae) (Table 3, Fig. 5).

**Table 3:** Coefficient estimates from linear mixed-effects models (LME) which included interaction terms to test whether temperature (Amb vs. + 2 °C vs. + 4 °C) and litter quality (natural vs. poor) influence the relationship between individuals alive and emergent adults of detritivore taxon (*Polypedilum* vs. Atalophlebiinae, *Polypedilum* vs. Calamoceratidae*,* and Atalophlebiinae vs. Calamoceratidae).

	Explanatory variable	Detritus consumption			
		Estimate	95%CI	75%CI	*p* value
*Polypedilum* versus Atalophlebiinae	Ind	− 0.019	− 0.81, 0.77	− 0.47, 0.43	0.959
${\text{R}}_{{{\text{marginal}}}}^{2}$ = 0.127	Ind * NatLitter Ambient	− 0.388	− 1.95, 1.17	− 1.2, 0.48	0.604
${\text{R}}_{{{\text{conditional}}}}^{2}$ = 0.892	Ind * NatLitter + 4 °C	− 0.359	− 1.64, 0.93	− 1.08, 0.36	0.561
	Ind * PoorLitter Ambient	− 0.308	− 2.80, 2.18	− 1.70, 1.09	0.795
	Ind * PoorLitter + 4 °C	1.186	− 0.55, 2.93	0.20, 2.16	0.168
*Polypedilum* versus Calamoceratidae	Ind	− 0.203	− 1.25, 0.84	− 0.79, 0.39	0.689
${\text{R}}_{{{\text{marginal}}}}^{2}$ = 0.07	Ind * NatLitter Ambient	− 1–165	− 3.27, 0.94	− 2.34, 0.01	0.256
${\text{R}}_{{{\text{conditional}}}}^{2}$ = 0.889	Ind * NatLitter + 4 °C	− 0.290	− 2.02, 1.44	− 1.26, 0.68	0.726
	Ind * PoorLitter Ambient	0.123	− 3.23, 3.47	− 1.76, 2.00	0.938
	Ind * PoorLitter + 4 °C	0.973	− 1.37, 3.32	− 0.34, 2.29	0.391
Atalophlebiinae versus Calamoceratidae	Ind	0.307	− 0.73, 1.35	− 0.28, 0.90	0.545
${\text{R}}_{{{\text{marginal}}}}^{2}$ = 0.118	Ind * NatLitter Ambient	0.437	− 3.21, 4.08	− 1.61, 2.48	0.801
${\text{R}}_{{{\text{conditional}}}}^{2}$ = 0.891	Ind * NatLitter + 4 °C	− 0.681	− 2.95, 1.59	− 1.95, 0.59	0.532
	Ind * PoorLitter Ambient	0.216	− 1.73, 2.16	− 0.87, 1.31	0.815
	Ind * PoorLitter + 4 °C	1.466	− 0.80, 3.73	0.19, 2.74	0.188
Emergent adults					
*Polypedilum* versus Atalophlebiinae		0.788	− 1.73, 0.17	− 1.32, − 0.23	0.104
${\text{R}}_{{{\text{marginal}}}}^{2}$ = 0.107	Adult * NatLitter Ambient	− 0.924	− 2.64, 1.32	− 1.89, 0.04	0.271
${\text{R}}_{{{\text{conditional}}}}^{2}$ = 0.889	Adult * NatLitter + 4 °C	− 1.218	− 4.41, 1.98	− 3.01, 0.57	0.429
	Adult * PoorLitter Ambient	− 0.846	− 2.61, 0.92	− 1.83, 0.14	0.323
	Adult * PoorLitter + 4 °C	0.136	− 2.61, 2.88	− 1.40, 1.68	0.917

Analysis was performed to individuals (Ind) alive and emergent adults separately. Confidence intervals are scaled. ${\text{R}}_{{{\text{marginal}}}}^{2}$ = variance explained only by fixed effects; ${\text{R}}_{{{\text{conditional}}}}^{2}$ = variance explained by fixed plus random effects.

## Discussion

Our study provides novel insights into the anticipated impact of current and projected increases in atmospheric CO_2_ concentration on detritivore performance and survival. This is particularly relevant in light of decreasing leaf litter quality and the interplay of species interactions amidst rising temperatures. We observed distinct responses of warming and CO_2_ concentration across different taxonomic levels, with some feeding strategies (e.g., collector-gathering by *Polypedilum*, and scraping by Atalophlebiinae) likely benefiting from living together^33^ under poor quality leaf litter. These findings suggest that detritus quality could mediate detritivore responses and species interactions to climate change. Our results suggest that, at community level, leaf litter quality and warming had a more pronounced impact on detritus consumption than on interactions among detritivores. Consequently, understanding the intricacies of how such interactions mediate specific responses at organismal and community levels become essential for a comprehensive understanding of the overall impact of climate change. Our findings highlight the complexity of these responses, emphasizing the need for future studies to investigate the underlying mechanisms and implications for ecosystem functioning in the face of climate change.

### Effects at organismal level

Previous research has demonstrated that poor-quality litter reduces the survival of *Polypedilum* and Atalophlebiinae^34–36^. Leaves with tough structures and high concentrations of recalcitrant carbon (C) content are less colonized by microorganisms and require longer conditioning time and biofilm formation^37,38^. The lack of biofilm formation in our study, which would benefit *Polypedilum* and Atalophlebiinae^39,40^, limits the access to nutritional resources and leads to decreased survival. Poor-quality litter also decreased the growth rate of Atalophlebiinae. Our results align with previous research, indicating that organisms experiencing limited resources exhibit slow growth, delayed maturity, and differential resource allocation towards maintenance (see^41^). However, the presence of molts and adult organisms in the poor-quality leaf litter treatment suggest that Atalophlebiinae may not only grow less but also reach maturity faster to reduce the risk of starvation. Therefore, deteriorating conditions can also act as a form of time constraint^41^, triggering fast maturation and development^41^. Contrary to previous studies^42,43^, poor-quality leaf litter did not affect the behavior or survival of Calamoceratidae. The range of lignin amount found on poor quality leaf litter treatment aligns with those found in the Mata Atlântica forest^42,44^, where our organisms inhabit. However, ongoing increases in CO_2_ emissions could alter leaf characteristics, potentially transforming riparian vegetation from a diverse leaf substrate to a more homogenized leaf substrate characterized by low nutrient content and a high percentage of carbon-based secondary compounds, with severe consequences for ecosystem functioning.

As expected, warming boosted the growth rate of *Polypedilum* and Atalophlebiinae and enhanced detritus consumption by Calamoceratidae. The most notable effect of temperature was observed at + 2 °C, remaining consistent at + 4 °C. Growth rates increase with rising temperature until reaching an optimum temperature, beyond which growth declines towards zero as thermal tolerance limits are approached^13,45,46^. The reduced behavior at + 4 °C suggests potential sublethal conditions for aquatic detritivores. Finally, poor-quality litter balanced out the positive impact of warming on the growth rate of *Polypedilum*, suggesting that litter quality can offset warming effects. However, the initially higher mortality of *Polypedilum* under poor-quality leaf litter complicates predictions regarding its response to warming. In our study, we observed different responses among taxon, which can be attributed to their feeding strategies, life cycles, and adaptability to changing conditions. Recognizing the complexity of these responses underscores the importance of considering the ecological dynamics and interplay within detritivore communities when assessing the potential impacts of climate change.

### Effects at community level

At the community-level, we found that changes in leaf litter quality and warming did not impact *Polypedilum* survival. *Polypedilum,* a collector-gatherer, relies less on leaf litter as a food source and more as a microhabitat. Furthermore, its short life cycle allows it to adjust quickly to varying conditions. Conversely, Atalophlebiinae showed increased nymph abundance in response to poor-quality leaf litter, likely due to its scraper behavior^39,40,47^ and the potential presence of biofilm on leaves generated by the presence of fungi. Fungi plays a crucial role in breaking down lignin and other carbon-based secondary compounds, making resources available to shredders and other scraper species^48^. Although leaves with higher lignin concentration may attract more fungi^48^, poor-quality litter had a negative impact on the abundance of Calamoceratidae. However, poor-quality litter did not affect Calamoceratidae adult emergence or its leaf litter mass consumption. Additionally, Atalophlebiinae, known for its facultative scraper behavior, can also function as a filtering-collector^49^, utilizing additional food resources such as organic matter from carcasses and dead organisms. Unlike Atalophlebiinae, Calamoceratidae seems to be vulnerable to changes in diet use, considering that it displays obligate shredder behavior, besides of being occasionally harmed by stoichiometric imbalances resulting from changes in detritus nutritional quality^37,50^. Our results suggest that when food quality changes, facultative detritivores can take advantage of other feeding sources, including carcasses and dead organisms, thus making them more resilient to changes caused by shifts in climatic conditions than species with obligate feeding strategies.

Warming counteracted the positive effect of poor-quality leaf litter, inhibiting the emergence of Atalophlebiinae and increasing detritus consumption. Given that growth is constrained by leaf litter nutritional quality^11,50^ and the increased energetic consumption caused by warming, we expected a growth limitation on poor-quality litter × warming treatment, which we observed with the reduction in the abundance of emerging adults and nymphs of Atalophlebiinae. The observed increase in the mass consumption of poor-quality leaf litter may be attributed to the effects of warming on energetic consumption. The increase in food intake, coupled with the reduced abundance of detritivores and emerging adults, suggests a compensatory feeding behavior. Conversely, warming independently increased the emergence of adults of Atalophlebiinae and decreased the abundance of Calamoceratidae larvae, suggesting taxon-specific responses. Climate change can alter species interactions, for instance, warming strengthened the intraspecific competition in the caddisfly *Limnephilus rhombicus*, resulting in reduced body mass and more efficient consumption of food sources^51^. Our warming treatment reduced the abundance of live larvae of Calamoceratidae. This suggests that warming strengthens interspecific interactions (i.e., competition), leading to a decrease in abundance and mass consumption. Given the contrasting responses observed at both the organismal and community level, as well as among different taxon, we suggest that both intra- and interspecific interactions contribute to how organisms respond to poor quality leaf litter and warming within the community.

### Species interactions and implications for ecosystems

Species interactions have significant implications for ecosystems. Although direct species interactions based on abundances were not detected (Fig. 5), intraspecific competition might have occurred within Calamoceratidae, while facilitative interactions benefits *Polypedilum* and Atalophlebiinae. Indeed, species interactions, such as competition and facilitation, play a significant role in shaping responses to environmental changes^52,53^. By facilitating leaf litter breakdown, Calamoceratidae impacts the entire community, particularly benefiting the smaller detritivores^54^. However, the decrease in Calamoceratidae abundance, likely influenced by intraspecific competition, might have resulted in reduced leaf litter breakdown, thus weakening facilitation interactions. Contrary to expectation, Atalophlebiinae and *Polypedilum* did not show negative impacts from poor-quality leaf litter, indicating their resilience to changes in Calamoceratidae abundance. The increased emergence of Atalophlebiinae adults, coupled with the absence of a significant response in larvae abundance, suggests that Atalophlebiinae might be leveraging alternative food resources, such as organic matter from decomposing organisms, to meet their nutritional demands for growth, survival, and metamorphosis. This strategy appears to be particularly important where leaf litter quality is poor. Additionally, the emergence of individuals from the aquatic ecosystem may alleviate resource competition^55^, positively impacting the survival of Atalophlebiinae nymphs. Furthermore, warming distinctly affected the abundance of specific taxonomic groups, with contrasting effects observed at both the organismal and community levels. Some species may face higher mortality due to overheating, while others might accelerate their development, thus emerging faster. This study suggests that interactions may strongly influence the response to warming, potentially modifying behavior, body size, or development rates. The complexity of ecological interactions highlights the need for comprehensive studies to determine the effects of climate change on detritivore communities and ecosystem processes.

In summary, our study revealed taxon-specific and scale-dependent responses to poor-quality leaf litter and warming among detritivores. Both intra- and interspecific interactions played a critical role in shaping these responses. Understanding these emergent properties is critical for accurately predicting the impacts of climate change on aquatic ecosystems. Further research is warranted to determine the underlying mechanisms driving these responses and to investigate their broader implications for ecosystem functioning in the context of ongoing climate change.

## Materials and methods

### Assessing leaf litter quality

#### Leaf litter CO_2_ enrichment

We maintained 60 plant seedlings of *Eugenia uniflora* L. (Myrtaceae) within two open-top chambers—OTC, each with a volume of 1.53 m^3^ each. These chambers were placed inside a greenhouse at the Section of Physiology and Biochemistry of the Botanic Institute of São Paulo, SP, Brazil. The construction details of the chambers, as well as the methods for CO_2_ injection and monitoring, were previously described by Braga et al.^56^. One OTC was subjected to elevated atmospheric CO_2_ concentration (892.04 ± 78.52 ppm), while the other was exposed to the natural CO_2_ atmospheric concentration (549.93 ± 19.31 ppm). Natural concentration of atmospheric CO_2_ is consistent with levels reported by Junior et al.^57^, for the city of São Paulo, SP, Brazil. The elevated CO_2_ concentration was based on the forecast for the year 2100 in the worst-case scenario, as outlined by the IPCC^1^. We conducted the experiment during the summer of 2017 (January–April), with a natural photoperiod of 12.5 h and a photosynthetic flux density at the surface of 400 mol m^−2^ s^−1^. The air temperature in the chamber varied from 32 to 18 °C (day to night) and the relative humidity varied from 51 to 70%. After three months, naturally abscised leaves of both CO_2_ treatments were collected, oven dried (60 °C, 72 h) for leaf chemistry characterization.

#### Leaf litter traits characterization

We assessed leaf litter quality by measuring the chemical composition of each leaf exposed to different levels of CO_2_. This analysis included: (i) C and N content; (ii) elemental ratios, determined as the initial carbon:nitrogen (C:N) ratios; (ii) concentrations of structural compounds, assessed as the initial percentage of lignin, and polyphenols; (iii) the ratio of the structural compounds to elemental content, providing an integrated, composite measure (lignin:N). Lignin:N ratio reflects the potential nutrient availability and the carbon quality of the leaf litter^58^. To measure nutrient concentrations, we grounded a subsample of leaf material pooled from 10 leaves per plant into a fine powder (~ 0.5 mm). C and N content were measured by dry combustion (1000 °C) in an Elementary Analyzer LECO TRuspec CHNS. We quantified lignin gravimetrically using the acid detergent fiber procedure described in Gessner^59^. To analyze polyphenols, we used UV/Visible Spectrophotometer (Mod. EW-83057-35). Leaf toughness was measured indirectly based on the force required to tear apart a leaf sample^59^. All analyses were conducted using 4 replicates.

To analyze the effect of CO_2_ enrichment on leaf chemistry traits, we conducted a permutational multivariate analysis of variance (PERMANOVA), using distance matrices (adonis function of the vegan package in R)^60^ and the Bray–Curtis dissimilarity coefficient; we used Monte Carlo permutation (999 permutations) to test the significance of the results. Subsequently, we performed a nonmetric multidimensional scale analysis (NMDS) to visually represent the similarity of leaf quality traits between the two CO_2_ treatments. For this, we assessed the stress and dimensionality “metaMDS” code^61^. Stress values below 0.05 indicate an excellent representation of the data, while stress values below 0.1 indicate good scaling with low tendency to error. Stress values exceeding 0.3 suggest that the points are placed arbitrarily in a two-dimensional classification^62^.

The elevated atmospheric CO_2_ concentration had a significant impact on leaf traits (PERMANOVA, R^2^ = 0.1595, *p* = 0.024, see Supplementary Material 1). However, there were no significant changes in the total C content (mean ± SD; natural = 46.24% ± 0.24, elevated = 45.43% ± 0.66, F_1.18_ = 1.28, *p* = 0.271) and polyphenol content (mean ± SD; natural = 37.49% ± 1.03, elevated = 40.63% ± 1.59, F_1.18_ = 2.74, *p* = 0.115) with increasing atmospheric CO_2_ concentration. Similarly, the total N content and the C:N ratio remained unaffected by the elevated CO_2_ levels (mean ± SD; N: natural = 2.30% ± 0.06, elevated = 2.16% ± 0.04, F_1.18_ = 3.44, *p* = 0.079; C:N: natural = 20.17 ± 0.46, elevated = 21.04 ± 0.40, F_1.18_ = 1.98, *p* = 0.176). In contrast, lignin content, a structural compound in plants, increased under elevated CO_2_ levels (mean ± SD; natural = 2.01% ± 0.18, elevated = 3.38% ± 0.44, F_1.18_ = 5.08, *p* = 0.036). This increase was further evident in the lignin:N ratio, which increased from 0.87 ± 0.08 under natural conditions to 1.57 ± 0.21 under elevated CO_2_ conditions (F1,18 = 9.32, *p* = 0.007). Additionally, leaf toughness was greater under elevated CO_2_ conditions (mean ± SD; natural = 266.9 g ± 13.12, high = 415.5 g ± 23.72, F_1.18_ = 30.03, *p* = 0.0005) (Supplementary Material 1)*.*

### Experimental design

#### Detritivore sampling

We conducted two experiments, the first at the organismal level and the second at the community level. The organismal-level experiment was conducted in the laboratory at the State University of Campinas, Campinas, Brazil. We used aquatic detritivores collected in three natural lakes in the Biological Reserve of Serra do Japi (23° 11′ S; 46° 52′ W), São Paulo state, Brazil (elevation ~ 1000 m). The community-level experiments were conducted in the laboratory of the Biological Reserve of Serra do Japi. In all experiments, we used the most common detritivore morphospecies: *Polypedilum* (Diptera: Chironomidae) as a collector-gathering, Atalophlebiinae (Ephemeroptera: Leptophlebiidae) as a scraper and facultative filtering-collector, and Calamoceratidae (Trichoptera) as a typical shredder. The climate in the study area is seasonal, characterized by a warm rainy season (November–April: 18–22 °C, 230 mm/month) and a cold dry season (May–September: 12–15 °C, 40 mm/month). The vegetation is characterized by semi-deciduous mesophytic Atlantic Rainforest^63^. The Biological Reserve of Serra do Japi is an important area for spring waters and hydric resources^64^. The ponds in this area are characterized by a mean pH = 4.88 ± 0.17, mean conductivity = 18.37 ± 2.57 μS cm^−1^, mean TDS concentration = 9.76 ppm ± 1.36, and mean oxygen saturation = 35.05% ± 2.56 (Benavides-Gordillo et al., unpublished data).

To collect the insects, we used a hand net with a mesh size of 150 µm. We conducted systematic sweeps through the bottom and water column to obtain a representative sample. We transferred the water and detritus collected to containers to triage and separated the detritivores organisms from the net content. We replicated the sampling process five times across the ponds. The individuals collected in the net were transported to the laboratory where we sorted the detritivores and stored them alive in 50 ml tubes. In the laboratory, we identified the insects to the lowest possible taxonomic level and recorded their abundances. The detritus was dried for 72 h at 60 °C to estimate their biomass, using an analytical scale (Ohaus Adventurer ARC 120,3100 g × 0.01 g precision). Finally, we calculated the density of detritivores (ind g^−1^ of detritus) for each sample, with the following means (± SD): *Polypedilum* 0.96 ind g^−1^ ± 0.43, Leptophlebiidae 0.28 ind g^−1^ ± 0.06, Calamoceratidae 0.04 ind g^−1^ ± 0.006.

#### Organismal-level experiment

To evaluate the impact of changes in leaf litter quality and warming on aquatic detritivores at the organismal level, including survival, emergence and leaf consumption rate, we used a full-factorial design (n = 90) consisting of two levels of leaf litter quality: (i) natural quality, leaf litter derived from plants grown under natural CO_2_ level, and (ii) poor quality, leaf litter derived from plants grown under elevated CO_2_ levels. These were combined orthogonally with three levels of temperature: (i) T_ambient_, (ii) T_ambient_ + 2 °C, the average projected for 2045 (IPCC, 2014), and (iii) T_ambient_ + 4 °C, the average projected for 2100 (IPCC 2014). Each microcosm received one of the following treatments: (i) natural-quality litter and T_ambient_ (control treatment); (ii) natural-quality litter and T_ambient_ + 2 °C; (iii) natural-quality litter and T_ambient_ + 4 °C; (iv) poor-quality litter and T_ambient_; (v) poor quality litter and T_ambient_ + 2 °C; and (vi) poor-quality litter and T_ambient_ + 4 °C (see Supplementary Material 2).

We individually reared morphospecimens of *Polypedilum*, Atalophlebiinae, and Calamoceratidae in three Bio-Oxygen Demand incubators (BOD, Eletrolab, model EL202), maintaining them at three distinct temperature levels: (i) T_ambient_; (ii) T_ambient_ + 2 °C; and (iii) T_ambient_ + 4 °C. In each BOD, we placed two transparent glass microcosms (volume = 50 ml) with two levels of litter quality: (i) natural-quality litter and (ii) poor-quality litter (Supplementary Material 2). The ambient temperature selected for the BOD experiment was determined based on the mean daily temperature observed in ponds during the same study season (mean ± SD: ambient = 20.51 ± 0.24, + 2 °C = 22.37 ± 0.20, + 4 °C = 24.24 ± 0.19 from October to December 2017). Each individual was fed with one leaf litter (mean ± SD: 0.09 ± 0.056 g) previously submerged by 48 h in mineral water to condition the leaves. The individuals of *Polypedilum* and Atalophlebiinae exhibited similar body size ranges with a length from 5 to10 mm), while the body size of Calamoceratidae ranged from 5 to 15 mm. At the end of the experiment, the microcosms were initially filled with mineral water. To ensure a consistent volume, we periodically supplemented them with distilled water as evaporation occurred. We used distilled water, to avoid additional nutrient sources.

We measured the body length of each insect to calculate their growth rate and weighted the leaf litter to estimate the leaf consumption rate at the beginning and at the end of the experiment (after 30 days). The growth and consumption rates were estimated as:

$$rate\;x = \frac{{x_{tf} - x_{t0} }}{{t\left( {days} \right)}}$$

where $x$ represents either both length ($t0$ estimating insect growth) or leaf mass (estimating detritus biomass loss through consumption), $t$ stands for time, *tf* represents the final time, and $t0$ denotes the initial time. To measure the length of each insect, we photographed each individual and measured their length using imageJ software^65^. Finally, we recorded the daily number of individuals that emerged as adults and those that died throughout the experiment (30 days).

#### Community-level experiment

To evaluate the impact of litter quality and warming on the community of aquatic detritivores (i.e., survival, emergence, and food consumption), we used a full-factorial randomized block design (n = 20). The experimental design included two levels of litter quality: (i) natural, leaf litter from plants grown under natural atmospheric CO_2_ concentration, and (ii) poor litter quality, leaf litter from plants grown under elevated atmospheric CO_2_ concentration. These were combined with two levels of water temperature: (i) T_ambient_ and (ii) T_ambient_ + 4 °C, the average projected for 2100. Due to the substantial number of microcosms, we performed the experiment in five blocks (see Supplementary Material 2).

We established a total of 20 glass aquariums, each with a volume of 1.5 L, to serve as microcosms. To ensure consistent conditions, we filled each aquarium with 1 L of filtered (with a mesh size of 150 µm) and homogenized water from three ponds, thereby eliminating all macroinvertebrate eggs and zooplankton. The substrate within each aquarium consisted of 2 g of leaf litter, previously conditioned by 48 h in filtered pond water, which had been cultivated under distinct atmospheric CO_2_ conditions: natural CO_2_ levels representing the quality of natural leaf litter, and elevated CO_2_ levels representing poor leaf litter quality.

To simulate warming conditions, we employed custom heating system of sensors and aquarium heathers (1 W, 110 V, Master) controlled by a central unit operating TotalControl Software® (Rio de Janeiro, Brazil) (see^16^). The heating setup consisted of a series of interconnected boxes housing the electronic components. Each individual component box was responsible for regulating and monitoring the temperature of four microcosms, with two maintained at the ambient temperature and the remaining two subjected to a 4 °C increase. To maintain a 4 °C above the ambient temperature of the unheated microcosms, we utilized submersible water heaters (1 W; n = 3) in half of the microcosms within each block. The temperature increase was achieved through submersible sensors within the unheated reference microcosms, which activated and deactivated the heater controllers in the heated microcosms. We conducted the experiment from October 3 to December 5, 2017.

In each aquarium, we introduced a specific number of individuals based on their natural densities (ind g^−1^ leaf litter) found in the ponds. Specifically, in each aquarium, we added five individuals of *Polypedilum,* two individuals of Atalophlebiinae, and two individuals of Calamoceratidae.

To mitigate potential losses of detritivores due to mortality or emergence during the experiment, we implemented a systematic introduction strategy based on our empirical observations regarding the resilience of each morphospecies to elevated temperatures. Every three days, two individuals of *Polypedilum* were introduced into each microcosm, while one Atalophlebiinae and one Calamoceratidae were added every seven days. By the end of the experiment, a total of 42 individuals of *Polypedilum*, 12 individuals of Atalophlebiinae, and 12 individuals of Calamoceratidae had been introduced into each microcosm.

Throughout the experiment, we monitored and recorded the emergence of adults. After 60 days, we collected all individuals and categorized them as either alive or deceased. Subsequently, we calculated the relative abundance of living individuals and emerging adults.

### Statistical analyses

#### Organismal-level experiment

We tested the effects of litter quality (two levels: natural vs. poor), warming (three levels: Ambient, + 2 °C, and + 4 °C), and their interaction on growth rate and leaf consumption rate by each detritivore taxon (Polypedilum, Atalophlebiinae, and Calamoceratidae) using generalized least square model (GLS) in the nlme package^64^. Because residuals were highly heteroscedastic, we modeled the variance using the function varIdent, using litter quality and temperature as the stratum. Since our experiment aimed to evaluate the individual response of each detritivore species to decreasing litter quality and warming, we conducted separate models for each species. To interpret models with significant interaction terms, post-hoc Tukey’s HSD tests were carried out using the multcomp package in R 3.1.3^66^.

We used survival-reliability analysis to compare the survivorship of each detritivore taxon in response to leaf litter quality and warming^67^. We tested the effects of leaf litter quality and warming using Cox's proportional hazard model. We conducted the survival-reliability analyses in JMP Pro version 17 (SAS 2022).

#### Community-level experiment

In this experiment, our aim was to evaluate the response of coexisting detritivore taxon to decreasing litter quality and warming. To achieve this, first, we used Multivariate Analysis of Variance (MANOVA) to test combined responses of three detritivores taxa to litter quality, warming and their interaction (litter quality × warming). We check all MANOVA assumptions, including multivariate outliers, multicollinearity, normality and homogeneity of covariances. Then, we used a linear mixed effect model (LME) with two fixed factors (leaf litter quality and temperature), which includes two levels of each factor (litter quality: natural and poor, and temperature: ambient and + 4 °C), their interaction, and a random factor (block) to test individual responses of each taxon. We used this model on the response variable: percentage of alive individuals, percentage of adults emerged, and leaf consumption rate in the treatments. As the detritivore taxa were coexisting, the rate of leaf consumption reflects the entire community and not each taxon individually. Finally, we tested the significance of fixed effects of the final model using a type III SS ANOVA. If the model validation plots showed heteroscedasticity, we implemented a variance function of the form varIdent (form =  ~ 1|litter × temperature), to estimate the within-group variance and account for unequal variances among the treatments.

Additionally, we evaluated whether the slope of the relationship between the number of individuals alive and leaf consumption rate varied in response to leaf litter quality and warming treatments. To achieve this, we added interaction terms for leaf litter quality × individuals alive, warming × individuals alive into the mixed effects models. We then assessed the estimated coefficients of these interactions on detritus consumption rate. To ensure comparability in parameter estimates from models, we scaled all the variables by dividing them by their standard deviations. Finally, we evaluated whether leaf litter quality (natural and poor), warming (ambient and + 4 °C), and their interaction influence the relationship among detritivore taxa. We used mixed-effect models to analyze the effects of leaf litter quality and warming on the relationships between the number of individuals alive of *Polypedilum* vs. Atalophlebiinae, *Polypedilum* vs. Calamoceratidae, and Atalophlebiinae vs. Calamoceratidae. We then evaluated whether the slope of such relationships changed across warming and leaf litter quality treatments.

## Data and code availability

All data generated and analysed for the current study are available on Figshare (10.6084/m9.figshare.25013426).

### Supplementary Information

Supplementary Information.
