# Supplementary material for: Warming and shifts in litter quality drive multiple responses in freshwater detritivore communities
Source: Sci Rep. 2024 May 15;14:11137. doi: 10.1038/s41598-024-61624-z (PMC11096378; doi:10.1038/s41598-024-61624-z)
Supplement: Supplementary file 1 — Supplementary Information. [file 41598_2024_61624_MOESM1_ESM.docx]

**Supplementary material**

**Warming and shifts in litter quality drive multiple responses in freshwater detritivore communities**

**Supplementary Material 1**

**Table 1**. Results of PERMANOVA of leaf traits from *Eugenia uniflora* plants grown under different concentrations of atmospheric CO_2_ (natural vs. high).

|  |  |  | **CO_2_ concentration** | | | | | | | | | | |
| --- | --- | --- | --- | --- | --- | --- | --- | --- | --- | --- | --- | --- | --- |
| *Source of variation* | |  | Mean | | |  | se | | |  | F |  | p |
|  |  |  | Natural |  | High |  | Natural |  | High |  |  |  |  |
|  | Carbon (%) |  | 46.24 |  | 45.43 |  | 0.24 |  | 0.66 |  | 1.28 |  | 0.271 |
|  | Nitrogen (%) |  | 2.30 |  | 2.16 |  | 0.06 |  | 0.04 |  | 3.44 |  | 0.079 |
|  | Lignin (%) |  | **2.001** |  | **3.38** |  | **0.18** |  | **0.44** |  | **5.08** |  | **0.036** |
|  | Polyphenol (%) |  | 37.49 |  | 40.63 |  | 1.03 |  | 1.59 |  | 2.74 |  | 0.115 |
|  | C:N ratio |  | 20.17 |  | 21.04 |  | 0.46 |  | 0.40 |  | 1.98 |  | 0.176 |
|  | Lignin:N ratio |  | **0.87** |  | **1.57** |  | **0.08** |  | **0.21** |  | **9.32** |  | **0.007** |
|  | Toughness |  | **266.9** |  | **415.5** |  | **13.12** |  | **23.72** |  | **30.05** |  | **0.0005** |

**
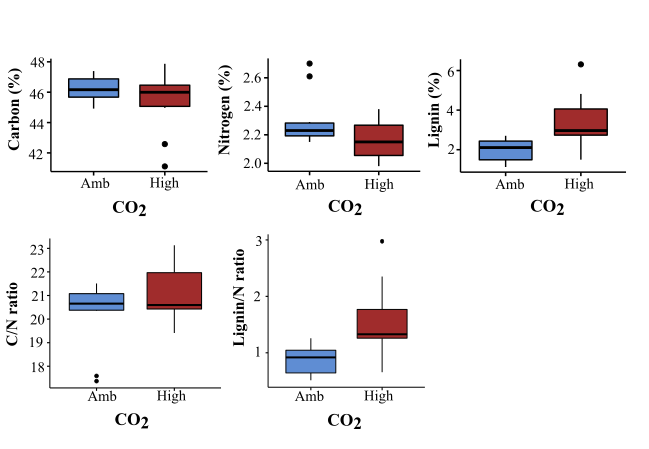
**

**Figure 1. Effects of increased atmospheric CO_2_ on leaf traits of *Eugenia uniflora*.** Effects of natural and increased atmospheric CO_2_ (high) on the percentage of carbon, nitrogen, lignin, and for the C:N and lignin/N ratios (n = 60). Box plots show the median (horizontal line), first and third quartile (rectangle), 1.5 × interquartile range (whiskers), and outliers (isolated points).


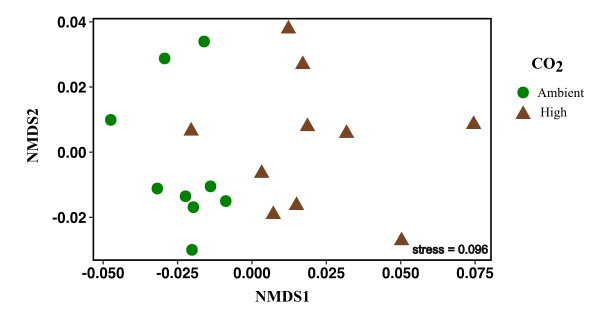


**Fig 2.** Non-metric multidimensional scaling (NMDS) of *Eugenia uniflora* leaf traits under two concentrations of atmospheric CO_2_ (natural vs. high). Stress value for the two main axes was 0.113.

**Supplementary Material 2**

**
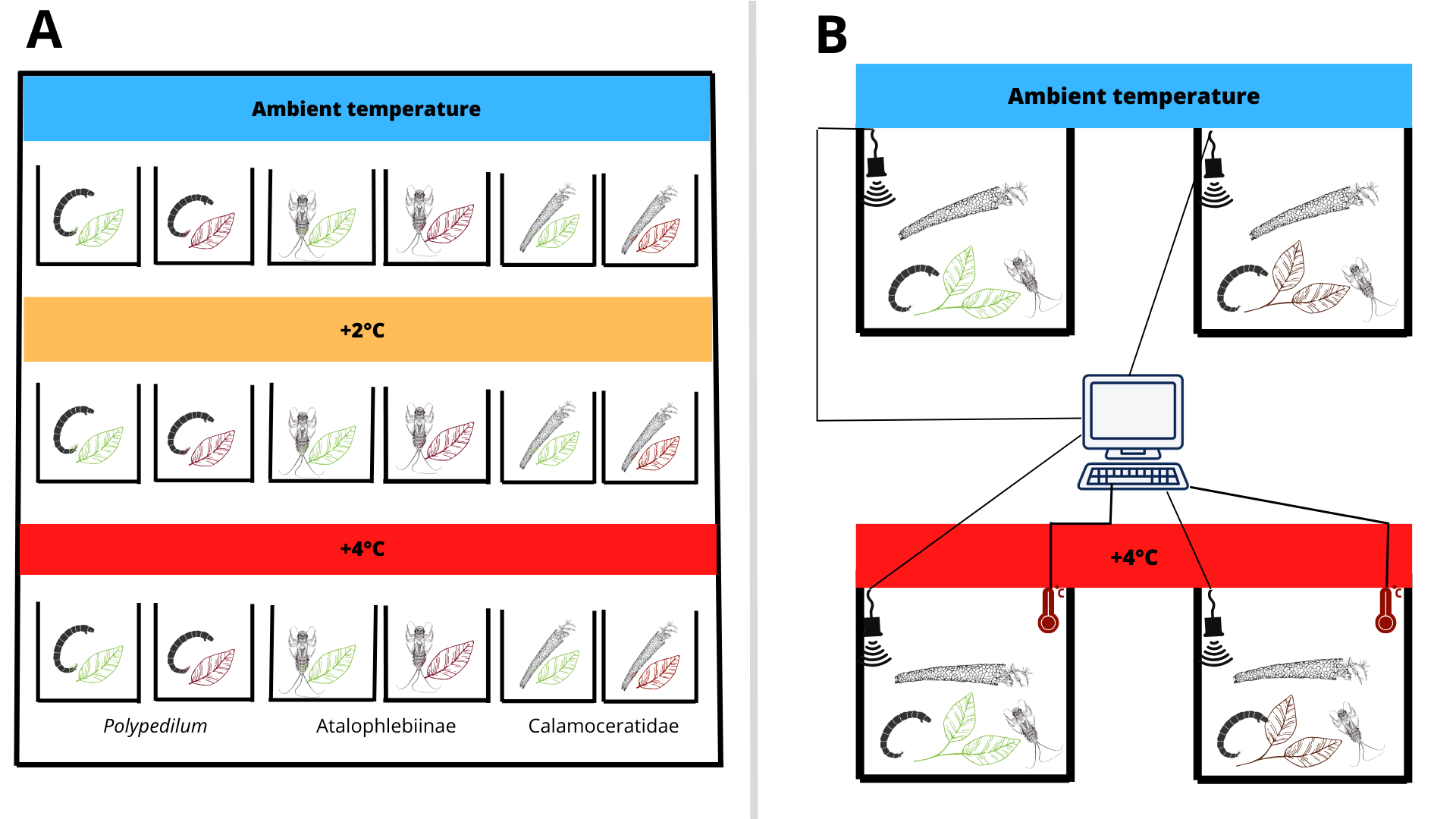
**

**Schematic diagram of the experimental design.** The leaf litter quality treatment was composed of two levels of leaf litter grown under different concentrations of atmospheric CO_2_: (i) natural-quality litter (green leaf) and (ii) average projected for 2100 (elevated atmospheric CO_2_ concentration, i.e., poor-quality litter) (brown leaf). The warming treatment was composed of three levels of temperature for the organismal-level experiment: (i) ambient temperature; (ii) average projected for 2050 (ambient +2^o^C); and (iii) average projected for 2100 under worst scenario (ambient +4^o^C) and two levels of temperature for the community-level experiment: i) ambient temperature; ii) average projected for 2100 under worst scenario (ambient +4^o^C). A) Incubators and BOD treatments (organismal level). Six treatment combinations with 5 replicates each (n = 30 microcosms); a) *Polypedilum*, b) Atalophlebiinae and c) Calamoceratidae. B) Schematic diagram of experimental design and temperature manipulation system used for the community-level experiment. The microcosms were divided into four treatments (natural litter and T_ambient_, natural litter and T+4^o^C, poor litter and T_ambient_, and poor litter and T+4^o^C). The temperature manipulation system consisted of heaters (in red) and temperature sensors (in black) connected to a computer.

**Supplementary Material 3**

**Table 1**. Coefficient estimates from linear mixed-effects models (LME) which included interaction terms to test whether temperature (Amb vs. +4^o^C) and litter quality (natural vs. poor) influence the relationship between detritivore diversity (percentage of individuals alive) and leaf consumption. Bolded confidence intervals denote those that did not overlap zero (i.e., statistically significant). Confidence intervals are scaled. R²_marginal_ = variance explained only by fixed effects; R²_conditional_ = variance explained by fixed plus random effects.

| **Detritus consumptions** | | | | | | | | | | |
| --- | --- | --- | --- | --- | --- | --- | --- | --- | --- | --- |
|  |  | Explanatory variable |  | Estimat |  | 95%CI |  | 75%CI |  | P-value |
| Individuals Alive |  | Larvae |  | 0.077 |  | **0.01, 0.13** |  | **0.04, 0.11** |  | 0.011 |
| R^2^_marginall_ = 0.415 |  | Larvae*NatLitter Ambient |  | -0.000 |  | -0.12, 0.12 |  | -0.07, 0.07 |  | 0.989 |
| R^2^_conditional_ = 0.927 |  | Larvae*NatLitter +4^o^C |  | 0.135 |  | -0.030, 0.30 |  | 0.04, 0.23 |  | 0.107 |
|  |  | Larvae*PoorLitter Ambient |  | 0.176 |  | **0.02, 0.32** |  | **0.09, 0.25** |  | 0.021 |
|  |  | Larvae*PoorLitter +4^o^C |  | 0.054 |  | -0.03, 0.14 |  | 0.004, 0.10 |  | 0.214 |

**Figure 1. Effects of warming and leaf litter quality on the relationship between individual’s survival and leaf consumption. a,** Relationship between relative detritivore survival and leaf consumption. **b,** Estimated coefficients for the interactive effects of detritivore survival on the leaf consumption in warming and litter quality treatments. Points represent scaled estimates, thick lines represent 75% CIs, and thin lines represent 95% CIs. All estimated and Cis were centered and scaled (by ones standard deviation to facilitate comparisons of effect sizes).
